# Algorithmic advances in smart TV content recommendation: a structured evidence-mapping review

**DOI:** 10.3389/fdata.2026.1927033

**Published:** 2026-09-14

**Authors:** Zhe Chen, Jing He, Yuanjia Gong, Junge Liang, Jilong Li

**Affiliations:** 1 State Key Laboratory of Media Convergence and Communication, Communication University of China, Beijing, China; 2 Academy of Broadcasting Science, National Radio and Television Administration, Beijing, China; 3 Department of Electronic Engineering, Hanyang University, Seoul, Republic of Korea

**Keywords:** evidence mapping, IPTV, large language models, multimodal recommendation, OTT, recommender systems, shared accounts, smart TV

## Abstract

Smart TV recommenders operate under constraints that are less prominent on personal devices: viewing is often passive, one account may represent several viewers, direct feedback is scarce, and programmes are long and semantically rich. We mapped research published from 2015 to 2025 using a structured evidence-review protocol. The submitted bibliography comprised 122 DOI-bearing references, including two foundational sources and 120 records used in the evidence map. Crossref verified 116 records; six stable arXiv DOI records were retained. Searches across six sources yielded 2,526 database-level results. A combined deduplication and title/abstract screening stage removed 2,414 records, leaving 112 unique records for full-text assessment; all 112 full texts were retrieved, 87 records were excluded, and 25 met the eligibility criteria. These 25 studies were combined with the 95-study initial corpus to form the 120-study evidence map. Separately, we conducted an independent dual-reviewer eligibility audit of 126 records, comprising all 120 included studies and six representative boundary exclusions. Agreement was 97.6% (123/126; Cohen's κ = 0.788). Six records were excluded by both reviewers, and three discordant judgments were resolved by joint full-record review. For analysis, each study was assigned one primary technical category and one of six mutually exclusive primary functional goals; secondary technical labels captured hybrid methods. Evidence reporting was profiled separately for data transparency, reproducibility, evaluation design, external validation, and deployment validation. The final literature corpus encompasses intelligent recommendation systems based on deep learning, sequence analysis, graph theory, shared accounts, and multimodal approaches.

## 1 Introduction

Smart television now encompasses linear channels, catch-up services, video-on-demand, apps, and commerce on the same connected screen. Unlike mobile use, television viewing often involves several people sharing a device and account. Recommendation therefore sits at the intersection of content discovery, household identity, and platform objectives such as engagement, revenue, and fairness (Alam et al., 2021; Tukuljac et al., 2015; Kovacik et al., 2017).

The recommendation problem is consequently different from its mobile and web counterparts. Remote controls offer little interaction, explicit ratings are unusual, and a single profile may represent viewers with conflicting preferences. Platforms must also recommend across live schedules, long-form programmes, and richly structured audiovisual content while serving several household members (Li et al., 2020; Alam et al., 2021; Alam and Khusro, 2020; Alam et al., 2019).

Existing reviews provide strong accounts of recommender methods, cold-start strategies, graph models, privacy, and multimodal learning (Javed et al., 2021; Ko et al., 2022; Gao et al., 2023; Chronis et al., 2024; Liu et al., 2024; Chen et al., 2025; Yin and Luo, 2021; Yin et al., 2025; Raza et al., 2024; Wang et al., 2023; Asad et al., 2023; Ge et al., 2025; Duricic et al., 2023; Jadon and Patil, 2025; Ping et al., 2025). Their conclusions do not transfer automatically to television. Shared accounts, passive viewing, live schedules, sparse feedback, and limited on-device resources alter both model design and evaluation.

Against this background, we ask four questions. RQ1 maps the distribution of studies published from 2015 to 2025 across technical paradigms and TV-specific functions. RQ2 examines which methods have credible advantages when signals are sparse, coupled, temporal, or multimodal. RQ3 considers the datasets and metrics needed to support household-aware and responsible television recommendation. RQ4 identifies gaps that remain between prototype results and evidence from deployed services.

The review makes three contributions. First, it verifies and organizes a corpus of 120 studies within an explicit television-relevant scope. Second, it cross-classifies technical paradigms against six mutually exclusive functional goals and supplements the primary counts with secondary technical labels that we verified for hybrid work. Third, it uses qualitative evidence domains to separate established findings from promising but weakly validated directions, including industrial systems and emerging uses of large language models.

Throughout the review, smart TV content recommendation refers to television-like viewing on smart TV, IPTV, OTT, video-on-demand, and live-TV services. We include work from adjacent domains only when it addresses a problem that transfers directly to this setting. Such problems include shared accounts, long-form video representation, multi-task objectives, privacy, and on-device inference.

## 2 Review methodology

### 2.1 Review design, protocol, and scope

We used structured evidence mapping rather than statistical meta-analysis because the included studies differ substantially in task definition, data provenance, candidate generation, negative sampling, metrics, baselines, and deployment setting. The review followed a structured evidence-mapping protocol aligned with the PRISMA 2020 framework and PRISMA-S search reporting guidelines. Methodological decisions, operational query strings, and multi-field coding criteria were established prior to formal synthesis (Table 1).

**Table 1:** Review protocol and reproducibility decisions.

Element	Operational decision
Time window	1 January 2015 to 31 December 2025 for the 120-study evidence corpus. Two older foundational references are retained only as background.
Search dates	We conducted the Google Scholar and IEEE Xplore searches on 1 April 2026 and the ACM Digital Library, Web of Science, Scopus, and Engineering Village searches on 1 May 2026.
Domain query	The core concept blocks cover television-like services plus recommendation or personalization. Supplementary Appendix S2 provides the 36 frozen conceptual variants and all 216 database-native search strings executed across the six databases, together with the recorded database-level result counts.
Challenge queries	Six domain phrases were crossed with six prespecified challenge blocks to form 36 frozen conceptual variants, all listed in Supplementary Appendix S2.
Sources	The six formal sources are Google Scholar, ACM Digital Library, Web of Science, IEEE Xplore, Scopus, and Engineering Village. The recorded database-level result counts were 604, 330, 392, 698, 356, and 146, respectively (total 2,526). After deduplication, mutually exclusive primary-discovery attribution among the 120 included records was 27, 18, 20, 39, 6, and 10, respectively.
Inclusion	English research from 2015–2025 with direct TV evidence or a clearly transferable method for one of the prespecified TV challenges; scholarly article, proceedings paper, review, or stable preprint.
Exclusion	Duplicates, non-recommender work, marketing-only discussion without algorithmic relevance, unverifiable records, and studies outside the date window. General studies without a direct transfer rationale were excluded.
Deduplication	Exact DOI matching, followed by normalized-title matching. Titles were lower-cased and stripped of punctuation for the second pass.
Evidence coding	Each record receives an evidence tier, one primary technical category, secondary technical labels where applicable, one of six mutually exclusive primary functional goals, metadata-verification status, evidence-source status, and reporting codes. Primary labels support mutually exclusive counts; secondary technical labels support overlap analysis.
Quality safeguards	Study credibility was appraised through separate domains rather than a composite score: design, data transparency, reproducibility, evaluation rigor, external validation, and deployment. At least one of us appraised every full text, and both of us checked a subset.
Independent review	After a randomized 20-record calibration exercise, Z.C. and J.H. independently assessed a separate 126-record eligibility-audit set comprising all 120 included studies and six representative boundary exclusions. Three discordant decisions were resolved by joint full-record review.
Agreement	Eligibility-audit agreement was 123/126 (97.6%), with Cohen's κ = 0.788. This coefficient evaluates inter-rater consistency within the 126-record validation cohort; it does not describe the earlier title/abstract stage or taxonomy coding.
Evidence basis	Each extracted field is linked to the source examined (full text, abstract, or metadata). Analyses based on different evidence levels use separate denominators.

We defined two evidence tiers before screening. TV-specific studies directly examined smart TV, IPTV, over-the-top, video-on-demand, live-TV, channel, or programme recommendation. Enabling or contextual studies were eligible only when they addressed a prespecified mechanism or evaluation problem that transfers directly to television, such as shared accounts, long-form content representation, temporal availability, privacy, or on-device inference. Enabling studies were not interpreted as direct evidence of effectiveness on television platforms.

### 2.2 Information sources and search strategy

The search strategy combined six television-domain phrases with six challenge blocks, yielding 36 prespecified conceptual query variants and 216 database-native executions across six formal sources: Google Scholar, ACM Digital Library, Web of Science, IEEE Xplore, Scopus, and Engineering Village. We conducted the Google Scholar and IEEE Xplore searches on 1 April 2026 and the remaining searches on 1 May 2026. The six sources yielded 2,526 database-level results: IEEE Xplore, 698; Google Scholar, 604; Web of Science, 392; Scopus, 356; ACM Digital Library, 330; and Engineering Village, 146. Initial title/abstract screening and cross-database deduplication were conducted as an integrated preliminary screening phase, removing 2,414 non-relevant or duplicate records and leaving 112 unique candidate publications for comprehensive full-text assessment. All 112 full texts were retrieved. Eighty-seven records were excluded because they lacked television-setting relevance or recommender-algorithm evaluation, and 25 met the eligibility criteria. We combined these 25 studies with the 95-study initial corpus to form the 120-study evidence map. (Khan et al., 2018; Li et al., 2015; Nixon et al., 2019; Gong et al., 2022; Pattanayak and Shukla, 2021; Cho et al., 2019; Maji et al., 2024; Li et al., 2018; Salampasis et al., 2023; Kim, 2020; Dudekula et al., 2023; Roy and Dutta, 2022; Javeed et al., 2024; Mansour et al., 2023; Lee et al., 2025; Esmeli et al., 2023; Zhang et al., 2016; Asabere and Acakpovi, 2020; dos Santos and Bianchini, 2015; Bae et al., 2023; Firth and Marinelli, 2025; Hu et al., 2019; Harichandan et al., 2025; Wang X. et al., 2016; Zhao et al., 2024; Yin et al., 2022b; Lu et al., 2015; Aisopos et al., 2017; Seo and Park, 2024; Véras et al., 2015; Seo et al., 2020; Cagali et al., 2023; Khan and Khusro, 2024; Kim et al., 2015; Mirbahar et al., 2025; Chang et al., 2024; Dang et al., 2021; Kharat et al., 2024; Yu et al., 2017; Yin et al., 2016; Abirami et al., 2023; Liu and Ren, 2023; Sarwar et al., 2001) and (Koren et al., 2009) serve as foundational background sources and are not included in evidence-map counts. Using a predefined database-priority sequence for cross-source attribution (IEEE Xplore → ACM Digital Library → Web of Science → Scopus → Engineering Village → Google Scholar), we assigned the 120 unique records to IEEE Xplore (39), Google Scholar (27), Web of Science (20), ACM Digital Library (18), Engineering Village (10), and Scopus (6). Supplementary Appendix S2 documents all 216 executed search strings, exact dates, and source-level yields; Supplementary Table S5 provides the screening and audit accounting; and Figure 1 summarizes corpus construction and the separate eligibility audit.

**Figure 1:**
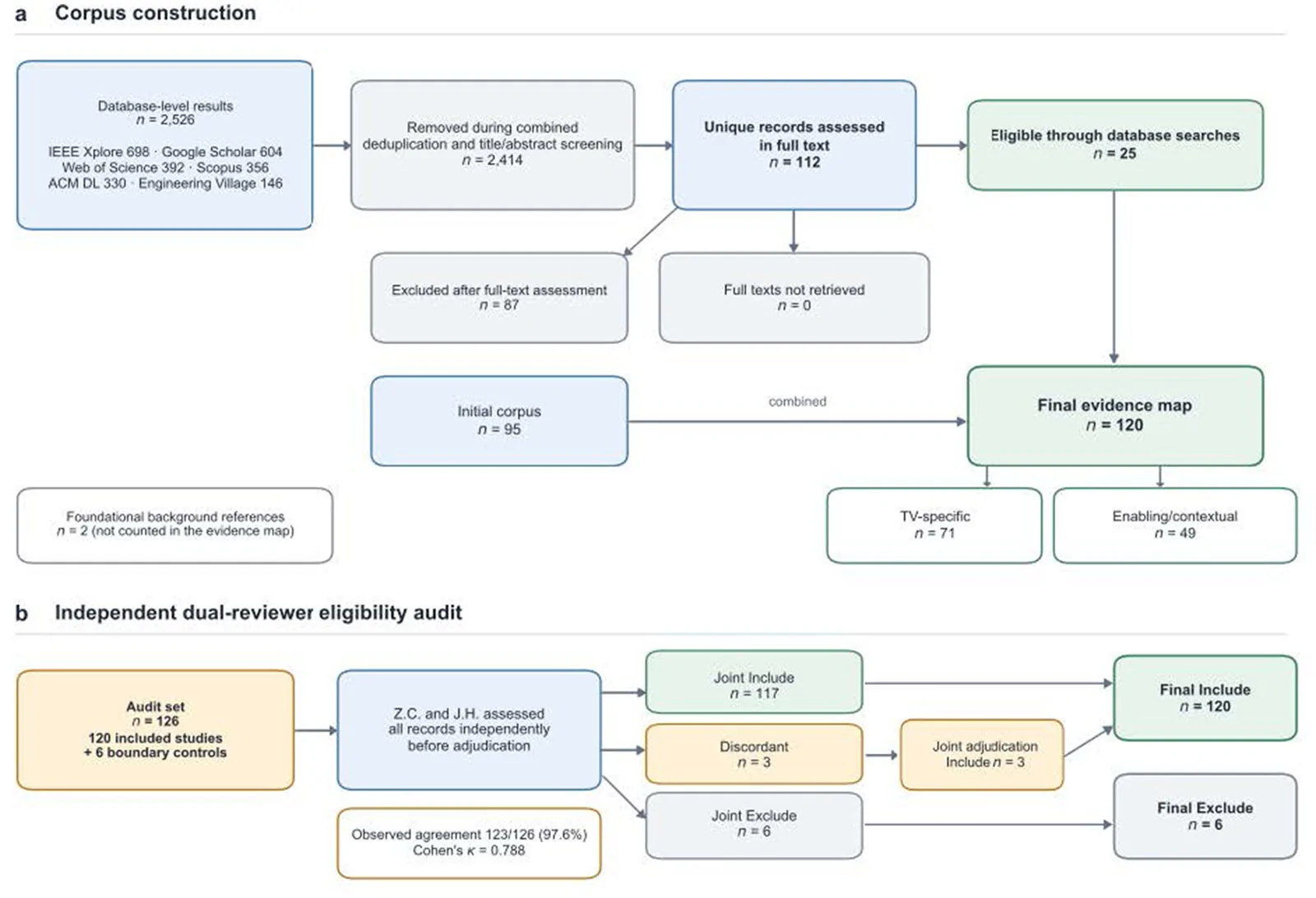
PRISMA-style accounting of corpus construction and the independent eligibility audit. **(a)** Searches across six sources yielded 2,526 database-level results. An integrated preliminary screening and deduplication phase removed 2,414 non-relevant or duplicate records. The remaining 112 unique records underwent full-text assessment; all full texts were retrieved, 87 records were excluded, and 25 met the eligibility criteria. These 25 studies were combined with the 95-study initial corpus to form the 120-study evidence map, comprising 71 television-specific and 49 enabling or contextual studies. Two foundational background references were retained outside evidence-map counts. **(b)** The separate 126-record eligibility audit comprised all 120 included studies and six representative boundary exclusions. Z.C. and J.H. assessed these records independently, producing 117 joint Include decisions, six joint Exclude decisions, and three discordant judgments. Joint adjudication classified the three discordant records as Include. Observed agreement was 123/126 (97.6%), with Cohen's κ = 0.788. The 126-record audit set is distinct from the 112-record database-search full-text set.

### 2.3 Eligibility criteria and record verification

Eligible records were English-language scholarly articles, proceedings papers, reviews, or stable preprints published from 1 January 2015 to 31 December 2025. Records required either direct evidence from a television-like setting or an explicit transfer rationale linked to a prespecified TV challenge. We excluded duplicates, non-recommender studies, marketing-only discussions without algorithmic or evaluative relevance, unverifiable records, out-of-window studies, and general recommendation studies without a direct transfer rationale. DOI and bibliographic metadata were verified through Crossref or OpenAlex, but registry verification was not treated as a substitute for eligibility screening.

### 2.4 Study selection and independent review

Before the eligibility audit, we (Z.C. and J.H.) evaluated a randomized pilot sample of 20 candidate records to calibrate the eligibility criteria and harmonize the operational codebook. We then conducted a separate independent eligibility audit of 126 records. The audit set comprised all 120 studies in the final evidence map and six representative boundary records selected from the excluded pool; it was distinct from the 112-record set assessed during database-search full-text screening. We assessed all 126 full texts independently without consulting one another's decisions. Before adjudication, Z.C. classified 119 records as Include and seven as Exclude, whereas J.H. classified 118 as Include, seven as Exclude, and one as Unclear. The contingency table contained 117 joint Include and six joint Exclude decisions, with three discordant judgments. Observed agreement was 123/126 (97.6%), and Cohen's κ was 0.788. The six jointly excluded records remained outside the evidence map because they addressed OTT adoption, video-service quality, smart-TV purchasing, IPTV cybersecurity, binge-watching behavior, or OTT usage intention without evaluating a recommender intervention. We jointly re-examined the three discordant records and adjudicated them as Include. The final audit outcome was therefore 120 included and six excluded records.

### 2.5 Data extraction, coding, and overlap analysis

Each included study received one primary technical category and one of six mutually exclusive primary functional goals for counting. Technical categories covered traditional or hybrid recommendation; deep, sequential, or multi-task modeling; graph and relational modeling; shared-account or group modeling; content understanding or foundation models; interaction or deployment systems; evaluation, trust, or responsible recommendation; and prior reviews. The six functional goals were sparse-feedback preference estimation, household disentanglement, content and semantic understanding, temporal and contextual adaptation, multi-objective and trustworthy recommendation, and deployment and interaction. Secondary technical labels were assigned where a study combined more than one methodological component.

The coding framework records one primary technical category and one of six primary functional goals for mutually exclusive counts, together with secondary technical labels for hybrid studies. We applied the frozen eligibility codebook during the independent 126-record eligibility audit and discussed ambiguous scope and transferability decisions during adjudication. We checked the primary and secondary classifications against the retrieved full texts and completed review records, then finalized them by consensus. The reported κ quantifies eligibility agreement within the 126-record audit set; it does not measure agreement at the title/abstract stage or category-specific coding reliability.

### 2.6 Critical appraisal and strength of evidence

A single quality score would have obscured rather than clarified the evidence because algorithm experiments, system descriptions, industrial case studies, reviews, and preprints use different designs. We therefore treated publication type as a descriptive stratum and profiled data transparency, method reproducibility, evaluation design, external validation, and deployment validation as separate evidence-reporting domains. At least one of us examined every full text, and both of us checked a subset before reaching consensus. These codes guided the narrative interpretation but were not used to rank papers or presented as a formal risk-of-bias instrument. The 120-study evidence corpus contains 68 journal articles, 39 proceedings papers, seven book chapters, and six stable preprints. The two foundational background references add one journal article and one proceedings paper but are not counted in the evidence map.

To maintain an empirical baseline across diverse publication formats, the structured evidence-reporting audit (Supplementary Table S4) categorized each study by its highest verified level of reporting. Studies that demonstrated algorithmic rigor exclusively on closed datasets or standard laboratory benchmarks without public artifacts, cross-dataset transfers, or live service integration were systematically characterized under baseline reporting strata (e.g., “Algorithmic prototype/Simulation-only,” “Single-dataset validation only,” and “Standard offline ranking”).

### 2.7 Evidence-source tracking and synthesis

Every extracted field is linked to the deepest source examined: full text, abstract, or bibliographic metadata. We retrieved the full texts of all 120 included records. At least one of us read each full text, and both of us independently checked a subset. Crossref supplied registry abstracts for 28 records, while the abstract-reporting audit used 90 available abstracts.

## 3 Evidence landscape

### 3.1 Publication trend and evidence tiers

The final evidence map contains 71 TV-specific studies and 49 enabling or contextual studies. To preserve the 120-record corpus without overstating its directness, every enabling record is accompanied by a television-transfer challenge and rationale. Separately from the six records excluded during the eligibility audit, six included studies (Jin et al., 2024; Gharibshah and Zhu, 2022; Rodríguez-López et al., 2024; Anute et al., 2024; Zhang, 2025; Yuwono and Sholihannisa, 2024) provide only weak boundary evidence. We retain these included boundary studies to document adjacent commercial, advertising, or cross-domain mechanisms, but do not treat them as validation of television recommendation systems.

### 3.2 Study credibility and evidence maturity

The full-text and metadata reporting profile showed that the 120-study corpus comprises 68 journal articles, 39 proceedings papers, seven book chapters, and six stable preprints. The two excluded foundational references comprise one additional journal article and one proceedings paper. Publication type was retained as a descriptive stratum rather than a quality signal. Interpretation drew on reported dataset access, reproducibility, comparative evaluation, external validation, and deployment evidence. This evidence-reporting profile distinguishes mature empirical support from exploratory results, system descriptions, reviews, and boundary evidence without imposing a single rank.

Figure 2 illustrates the overall publication trajectory and structural distribution across the verified 120-study evidence corpus. Panel a presents the annual publication counts stratified by evidence tier, while panel b delineates the corpus across the eight primary technical categories. Exact mutually exclusive counts are given in Table 2. Traditional and hybrid methods form the largest group; evaluation and responsible recommendation remain sparsely represented.

**Figure 2:**
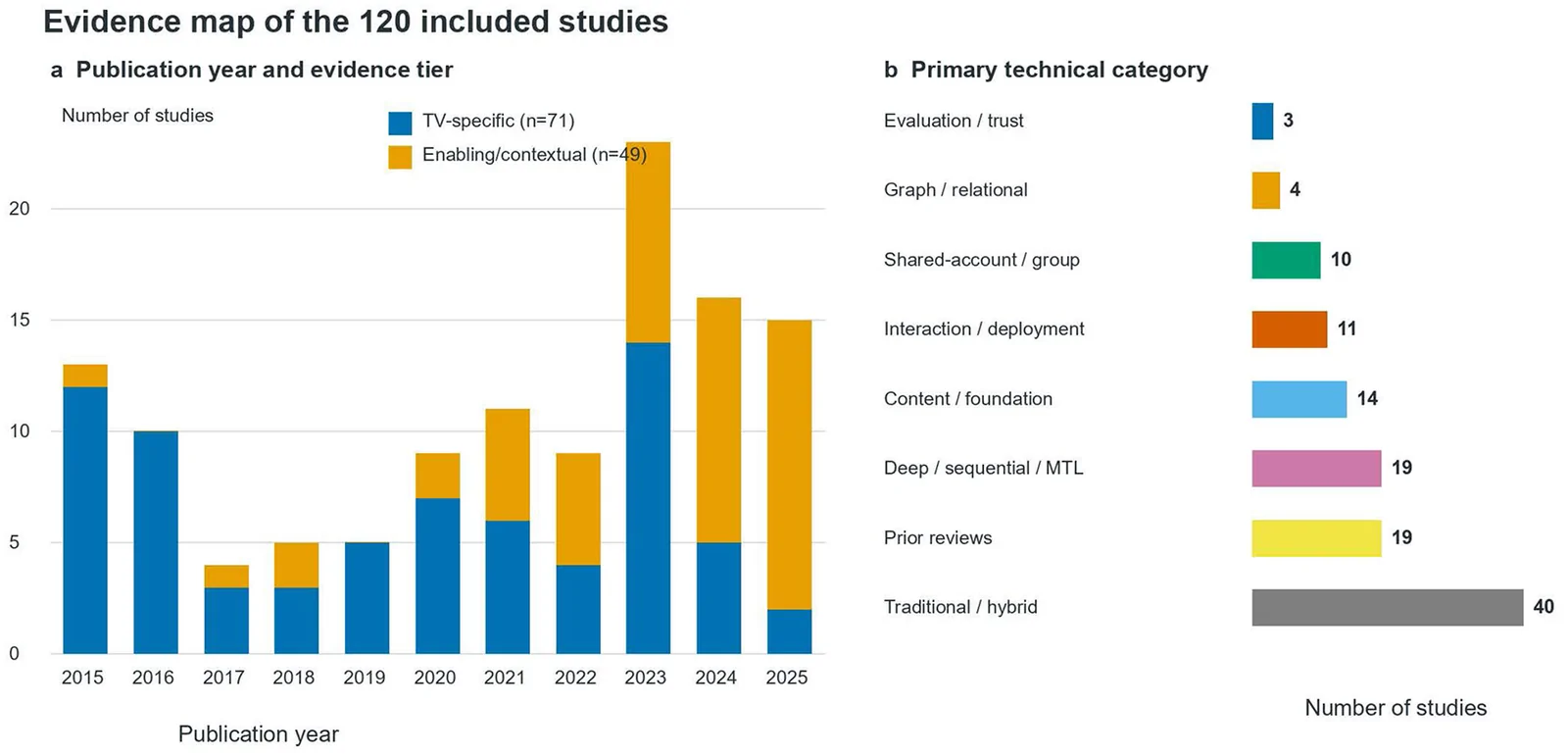
Evidence map of the 120 included studies. **(a)** Annual publication counts are stacked by evidence tier. TV-specific evidence directly concerns smart TV, IPTV, OTT, video-on-demand, or live-TV recommendation. Enabling/contextual evidence addresses transferable mechanisms. **(b)** Each study is assigned one primary technical category for counting. Counts reflect the verified corpus and do not represent effect sizes or publication quality.

**Table 2:** Distribution of included studies by primary technical category.

Primary category	All	TV-specific	Enabling	Main focus
Traditional and hybrid recommendation	40	29	11	CF, content, tensor, temporal, and hybrid baselines
Deep, sequential and multi-task models	19	15	4	Attention, transformers, long-short interests, MTL
Graph and relational recommendation	4	4	0	Heterogeneous, tripartite, and graph collaborative models
Shared-account and group modeling	10	8	2	Viewer identification, role separation, group aggregation
Content understanding and foundation models	14	7	7	Multimodal features, semantics, LLMs, MLLMs
Interaction and deployment systems	11	6	5	EPG, zapping, on-device, platform and field systems
Evaluation, trust and responsible recommendation	3	0	3	Fairness, privacy, explainability, beyond-accuracy goals
Prior reviews and field synthesis	19	2	17	General and domain-specific synthesis

### 3.3 Interpretation of the distribution

Older and newer approaches coexist rather than replacing one another. Traditional and hybrid designs remain useful when logs are sparse, computation is constrained, or decisions must be traceable. A time-aware optimisation method, for example, remained competitive on a standalone television while maintaining low latency (Kim et al., 2018).

Deep models are used chiefly for implicit sequences, auxiliary information, and attention (Li et al., 2020; Yin et al., 2023a,b, 2022a; Zhu et al., 2020; Zhou et al., 2021; Li et al., 2021; Yin et al., 2022c; Wu et al., 2024; Yang et al., 2018). Graph models appeared later and represent heterogeneous relations among users and programmes (Li K. et al., 2025; Yin et al., 2023c; Yao et al., 2023; Zhang et al., 2023). Both families demand more memory and costlier training or updating, yet few studies test whether those costs are acceptable on television hardware.

Household coupling is central to television use, but dedicated shared-account studies remain uncommon. COSMO combines session-aware co-attention with device information as weak supervision and reports offline and online results (Qin et al., 2023). PSJNet instead learns role-specific behavior across domains (Sun et al., 2023). Neither provides a shared household benchmark or a common treatment of consent.

Evidence on foundation models remains preliminary. The relevant literature includes multimodal representation, semantic annotation, and recent LLM-based work (Liu et al., 2024, 2025; Malitesta et al., 2025; Zhang et al., 2017; Ferreira et al., 2020; Raj et al., 2024). Direct evaluation of LLMs for movie or television recommendation is rare, and production evidence from smart-TV interfaces is rarer still.

## 4 Domain-specific challenges and research synthesis

### 4.1 Sparse input and preference ambiguity

Television studies rely heavily on implicit feedback because explicit ratings interrupt lean-back viewing. Yet a click or a long session is not an unambiguous preference label. A programme may continue after a viewer leaves, another person may take the remote, and a live schedule may remove alternatives. Treating all exposure as deliberate preference can therefore reinforce popularity and favor the household majority.

Sequential and contextual models can reduce part of this ambiguity. The IPTV Transformer combines implicit histories through a fusion layer (Li et al., 2020), while DeepTV adds electronic programme guide features for live-TV cold start (Zhu et al., 2020). Tensor and quadratic-programming methods explicitly represent time context (Kim et al., 2018; Yin et al., 2018). Each reports improved relevance in its own setting, but differences in data and baselines rule out a defensible ranking across studies.

Evaluation needs to separate exposure from active selection, dwell, completion, replay, abandonment, and remote-control events. Failure to watch should not be coded as negative feedback when a programme was unavailable or never displayed. Propensity correction and counterfactual methods are especially relevant when the interface itself controls exposure. Table 3 links these television-specific problems to representative responses and remaining evidence gaps.

**Table 3:** TV-specific challenges, representative responses, and unresolved evidence gaps.

Challenge	Why it is TV-specific	Representative responses	Unresolved issue
Sparse and ambiguous input	Remote controls and passive viewing produce few explicit judgements. A long view may indicate interest, inertia, or co-viewing.	Implicit sequences, EPG features, viewing duration, temporal context, and attention (Li et al., 2020; Zhu et al., 2020; Li et al., 2021; Yang et al., 2018; Kren et al., 2019; Yin et al., 2018).	Reliable counterfactual labels and low-burden explicit feedback remain under-tested.
Shared household identity	One account mixes several people and groups. The active viewer may change within a session.	Demographic group profiles, viewer recognition, latent role separation, weak device supervision, and split-join networks (Haq et al., 2021; Alam and Khusro, 2020; Qin et al., 2023; Wang and He, 2016; Zhu et al., 2023; Iguchi et al., 2015; Zhou, 2015; Sun et al., 2023).	Identity error, consent, children, guests, and fairness across household members lack common evaluation.
Long-form content semantics	Narrative, audio, cast, topic, mood, and episode structure unfold over long durations.	Metadata fusion, semantic annotation, visual/audio features, knowledge graphs, and multimodal or language models (Jan et al., 2022; Liu et al., 2024; Yin et al., 2023b,c; Yao et al., 2023; Yang et al., 2024; Malitesta et al., 2025; Zhang et al., 2017; Gao et al., 2015; Ferreira et al., 2020; Raj et al., 2024).	Temporal grounding, spoiler control, multilingual content, and efficient feature refresh remain open.
Temporal and live-TV constraints	Programme availability, schedules, remaining time, and channel-switching costs shape utility.	Time-aware preferences, tensor decomposition, dynamic interests, contextual ranking, and personalized EPGs (Yin et al., 2023a; Tukuljac et al., 2015; Li et al., 2021; Kim and Kim, 2016; Adeliyi et al., 2021; Kim et al., 2018; Liu P. et al., 2016; Yang et al., 2015; Aharon et al., 2015; Yin et al., 2018).	Many evaluations ignore real-time candidate changes, zapping delay, and household interruptions.
Competing objectives	Accuracy, watch time, satisfaction, diversity, revenue, fairness, privacy, and latency may conflict.	Multi-task routing, explanations, beyond-accuracy objectives, and privacy-preserving learning (Asad et al., 2023; Ge et al., 2025; Duricic et al., 2023; Jadon and Patil, 2025; Ping et al., 2025; Yin et al., 2021, 2023d; Tang et al., 2020; Zheng and Wang, 2021; Li X. et al., 2025).	Objective weights and trade-offs are rarely validated with household-level outcomes.

### 4.2 Household coupling and group utility

A household recommender must do more than average individual preferences. It must infer who may be present and decide how competing preferences should be combined. Demographic recognition can estimate age or gender proxies (Haq et al., 2021; Alam and Khusro, 2020), but camera-based sensing raises privacy and robustness concerns. Log-based individualization avoids direct sensing, yet may confuse similar viewers or fail when a new household member appears (Wang and He, 2016; Iguchi et al., 2015).

More recent models draw on session context and weak supervision. COSMO jointly attends to candidate items, the current session, and mixed viewing histories (Qin et al., 2023). PSJNet learns role-specific representations from shared-account sequences across domains (Sun et al., 2023). Both approaches recast the task as uncertain role inference rather than fixed profile estimation.

Ranking accuracy alone cannot reveal the household consequences of these choices. Useful measures include active-viewer identification, worst-member satisfaction, sensitivity to disagreement, exposure across inferred members, and robustness to mistaken identity. Interfaces should also make inferred identity visible and easy to correct.

### 4.3 Content semantics and temporal structure

Metadata-based methods are efficient, but incomplete catalogs and narrative complexity limit what they can represent. Multisource neural models combine tags, titles, and heterogeneous features (Yin et al., 2023b, 2022a; Wu et al., 2024). xLiMe links live television with news and social streams across languages and modalities (Zhang et al., 2017). VecLP integrates visual and textual information from live broadcasts (Gao et al., 2015), while the Globoplay study examined perceived audiovisual similarity in user interviews (Ferreira et al., 2020).

Multimodal representations may improve cold-start retrieval, but they also introduce distinct errors. Models can overfit trailers, subtitles, or promotional images, while programme-level aggregation can hide important episode-level events. Generated summaries may disclose spoilers or invent attributes. Deployment therefore depends on traceable source data, event-level segmentation, controlled updates, and cached representations that meet serving constraints.

### 4.4 Explicit feedback beyond recognition of the constraint

Among the 90 available abstracts, 17 mentioned explicit feedback and 16 mentioned implicit feedback. These are terminology counts, not adoption rates. In practice, TV-specific studies still rely mainly on viewing logs, ratings collected outside the immediate session, or inferred preferences (Li et al., 2020; Pyo et al., 2015; Alam and Khusro, 2020; Zhu et al., 2020; Kren et al., 2019; Yin et al., 2018).

Television interfaces need not copy mobile questionnaires to collect feedback. A one-button response, brief viewer switch, voice confirmation, or two-choice prompt after viewing may be less intrusive. Explanations can also invite corrections such as “less like this.” Such prompts should remain optional, infrequent, and reversible, with non-response treated as unknown rather than negative.

Whether these mechanisms improve recommendations without disrupting viewing remains uncertain. Longitudinal studies should track response, abandonment, accessibility, unequal household participation, and the persistence of corrected preferences. The value of explicit feedback depends on both its acquisition cost and whose preferences it represents.

## 5 Technical taxonomy and comparative assessment

Figure 3 cross-classifies every study by one primary technical paradigm and one of six mutually exclusive primary functional goals. We report the verified secondary technical-label frequencies and pairwise co-occurrence counts in Supplementary Table S6. Across the corpus, the most frequent secondary technical labels were interaction or deployment systems (*n* = 21), traditional or hybrid recommendation (*n* = 17), and deep, sequential, or multi-task models (*n* = 15). These multi-label counts capture hybrid methods without altering the 120-study primary denominator.

**Figure 3:**
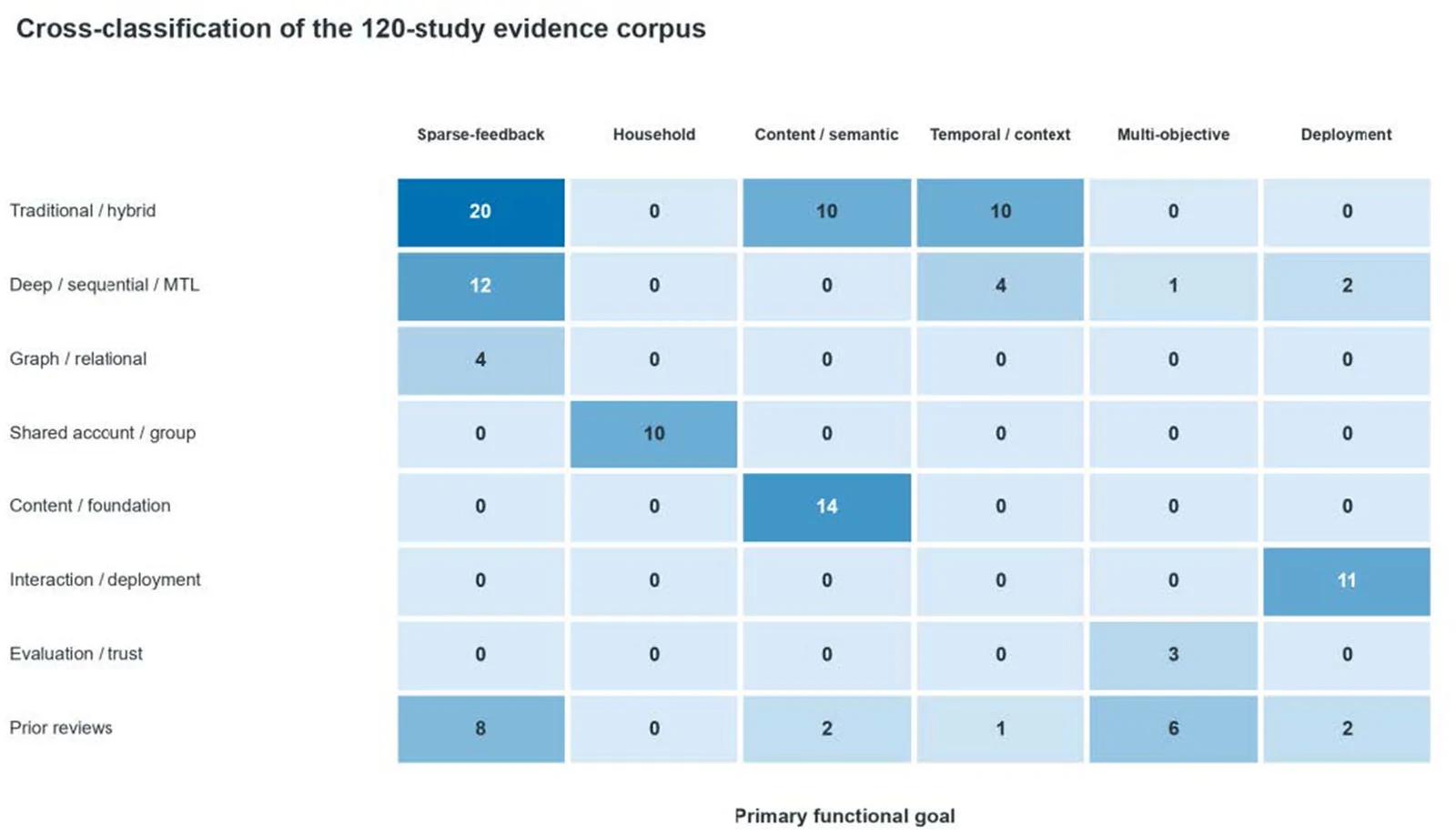
Cross-classification of primary technical categories and six mutually exclusive primary functional goals in the evidence corpus. Rows represent mutually exclusive primary technical categories and columns represent mutually exclusive primary functional goals; each of the 120 studies appears once. We report the verified secondary technical-label frequencies and pairwise co-occurrence counts for hybrid methods in Supplementary Table S6. The matrix describes research emphasis, not comparative performance or publication quality.

### 5.1 Traditional, context-aware, and hybrid methods

Collaborative filtering and matrix factorization remain useful baselines because they are efficient and interpretable (Sarwar et al., 2001; Koren et al., 2009). Their assumptions are strained in live television, where programmes change quickly, feedback is sparse, and one account may represent several viewers. Content and context features address some of these constraints (Pyo et al., 2015; Avila et al., 2015; Tukuljac et al., 2015; Wang, 2023; Kim et al., 2018; Liu P. et al., 2016; Chang et al., 2016; Yang et al., 2015; Aharon et al., 2015; Wang F. et al., 2016; Vieira and Lino, 2015; Shanmugalingam et al., 2020; Yin et al., 2018; Zhou, 2015; Liu J. et al., 2016).

Traditional and hybrid methods are often the most practical choice when data and computation are limited. A time-aware quadratic programme designed for a low-power standalone television outperformed its reported baselines (Kim et al., 2018). Hybrid semantic and data-mining systems can also improve cold-start coverage (Vieira and Lino, 2015), although they depend on hand-designed features and continued maintenance.

### 5.2 Deep, sequential, and multi-task models

Deep models capture nonlinear interactions and changes in sequential preference. Attention and Transformer variants use programme sequences, heterogeneous attributes, and short-and long-term interests (Li et al., 2020; Yin et al., 2023a,b, 2022a; Zhou et al., 2021; Li et al., 2021; Yin et al., 2022c; Wu et al., 2024; Yang et al., 2018). These gains in capacity come with expensive retraining, difficult calibration, limited interpretability, and sensitivity to exposure-biased labels.

Multi-task learning suits platforms that optimize clicks, views, watch time, and retention simultaneously. PLE separates shared and task-specific components and produced online gains on a large video platform (Tang et al., 2020). That result supports deployment in general video recommendation, but does not determine appropriate objective weights for households or public-service media.

### 5.3 Graph and relational recommendation

Graph models encode relations among users, programmes, labels, actors, channels, and contexts (Li K. et al., 2025; Yin et al., 2023c; Yao et al., 2023; Zhang et al., 2023). They can transfer information to cold items and support relational explanations. Their practical costs include graph construction, memory, stale edges, and expensive incremental updates. Current TV-specific evidence is too limited to establish a general advantage over other approaches.

Graph recommendation is most attractive when relations are reliable and reusable. Its value declines when metadata change rapidly or household identity remains uncertain. Comparisons should therefore report preprocessing and serving costs alongside ranking metrics.

### 5.4 Representative method comparison

Table 4 compares representative approaches in terms of evidence and data, evaluation design, computational or deployment complexity, principal strengths, and limitations.

**Table 4:** Comparative assessment of representative approaches.

Approach	Evidence and data	Evaluation	Complexity/ deployment	Strength	Limitation
Unified LDA topic model Pyo et al. (2015)	7 months of real viewing and EPG data	Precision; 81.4% average for 50 topics	Moderate; interpretable latent topics	Combines user and programme semantics; helps item ramp-up	Topic granularity and historical evaluation may not transfer
IPTV Transformer fusion Li et al. (2020)	Real IPTV application plus two open datasets	Offline comparison; production effectiveness stated	High training cost; feasible server-side	Models mixed implicit sequences	Account coupling remains latent; limited public reproducibility
DeepTV Zhu et al. (2020)	Industrial live-TV logs and EPG	Offline improvement over baselines	Moderate to high	Models availability, remaining time, and cold start	Private data; cross-platform generalization unknown
COSMO Qin et al. (2023)	Commercial TV logs with device information	Offline experiments and online A/B tests	High; server-side attention model	Weak supervision for mixed household behavior	Requires informative device/session structure
Group profile generation Alam and Khusro (2020)	Prototype; demographics and ratings	Recommendation and satisfaction analysis	Moderate; optional camera sensing	Anonymous group profile and dominant-member logic	Demographic proxies and sensing raise fairness/privacy issues
Time-aware quadratic model Kim et al. (2018)	Real-world TV dataset	Reported 5%−50% improvement over baselines	Low; designed for standalone TV	Explicit temporal conflicts and device feasibility	Restricted feature family; dataset not public
Heterogeneous graph models Yin et al. (2023c); Yao et al. (2023)	TV interactions plus auxiliary information	Offline ranking comparison	High memory and update cost	Uses multi-relational structure	Serving cost and online impact not established
PLE multi-task model Tang et al. (2020)	Tencent video; one-billion-sample training set	Offline plus online: +2.23% views, +1.84% watch time	High; deployed at platform scale	Reduces negative transfer between tasks	General video evidence; household utility not measured
Globoplay multimodal study Ferreira et al. (2020)	Platform catalog and user interviews	Perceived similarity; A/B test proposed	High offline encoding; low cached serving cost	Direct industrial multimodal case	No production business impact reported
Industrial VoD pipeline Bernardis et al. (2022)	Raw logs from an industrial provider	Next-item accuracy across session settings	End-to-end data engineering required	Exposes cleaning, sessionisation, and deployment constraints	Private data; business and fairness outcomes limited
PSJNet Sun et al. (2023)	Simulated Amazon and real commercial smart-TV logs	MRR and Recall	High; cross-domain sequence model	Separates roles and transfers cross-domain signals	Simulation assumptions and synchronous domains limit transfer
LLM movie discovery Raj et al. (2024)	MovieLens-100K and trailer subtitles	Ranking metrics against supervised baselines	Very high unless distilled or cached	Connects language semantics with preference history	Movie benchmark; no smart-TV online validation

## 6 Datasets and evaluation

### 6.1 Dataset landscape and reproducibility

Data access remains a central constraint. Only two of the 90 available abstracts named a public dataset, whereas 16 referred to proprietary, commercial, field, or real-world logs. This conservative terminology audit shows that abstracts rarely foreground provenance. Proprietary logs capture realistic exposure and sessions, but restrict independent replication.

Public movie datasets support controlled ranking experiments, yet omit household identity, remote-control events, live availability, zapping, and co-viewing. Simulated shared accounts can test role separation (Sun et al., 2023), although results depend on how individual histories are merged. A stronger benchmark suite would combine public movie data, de-identified television sessions, and a transparent household-simulation protocol.

MovieLens sample sizes follow the official release descriptions. For proprietary studies, “not reported” indicates that the original publication did not state the corresponding numerical quantity. Table 5 summarizes representative datasets, sample characteristics, availability, and limitations.

**Table 5:** Representative dataset types used or needed in smart TV recommendation.

Dataset/source	Users	Items	Interactions/ context	Availability	Best use and limitation
MovieLens 100K	943	1,682	100,000 explicit ratings	Public	Transparent ranking baseline; lacks TV sessions and households. Used in recent LLM movie work (Raj et al., 2024).
MovieLens 1M	6,040	3,706	1,000,209 ratings	Public	Larger explicit-feedback baseline; dated catalog and no exposure logs.
MovieLens 25M	162,541	62,423	25,000,095 ratings and tags	Public	Scale and content metadata; no TV interaction constraints.
7-month TV and EPG logs Pyo et al. (2015)	2,032	1,108 programmes analyzed from 4,313 original programmes	7-month viewing histories and EPG data across 20 channels	Proprietary	Real programme dynamics; limited independent reproduction.
DeepTV industrial logs Zhu et al. (2020)	500 experimental accounts sampled from logs of 35,143 set-top boxes	Live programmes; item count not reported in the original publication	30,187,636 viewing records (17 May-20 June 2017), EPG, remaining time, and positive/negative signals	Proprietary	Cold-start and live availability; provider-specific exposure.
Commercial COSMO logs Qin et al. (2023)	Numerical account count not reported in the original publication	TV content	Mixed account histories, sessions, devices, online outcomes	Proprietary	Household coupling and A/B tests; access and privacy constraints.
Globoplay study Ferreira et al. (2020)	Qualitative study group size not reported in the original publication	Platform catalog	Audio, visual, and perceived similarity	Proprietary	Industrial multimodal relevance; no reported online impact.
PSJNet datasets Sun et al. (2023)	260,000 HVIDEO users; HAmazon accounts simulated by merging 2–4 users	Movies/books and commercial TV content	Simulated and real shared-account cross-domain sequences	Mixed; public-source and proprietary	Role separation; simulation and domain assumptions matter.

For proprietary studies, “not reported” means that the corresponding numerical quantity was not reported in the original publication.

### 6.2 Abstract-level reporting audit

The separate abstract-reporting audit found that 16 of 90 abstracts named at least one evaluation metric. Precision appeared in six abstracts and Recall in four; Diversity and Fairness each appeared in three. Serendipity, CTR, Coverage, MAP, AUC, F1, Novelty, RMSE, MAE, and MRR each appeared once. Counts are not mutually exclusive because one abstract may name several metrics.

### 6.3 Evaluation design priorities

Offline evaluation should use temporal splits to prevent future information from entering training data. Candidate sets also need to reflect programme availability. In shared-account experiments, role identification and ranking require separate evaluation; otherwise, gains may be attributed to the wrong component.

Online experiments need a prespecified primary outcome and appropriate guardrails. More watch time is not necessarily beneficial if abandonment or zapping increases, exposure narrows, or minority household preferences are suppressed. Longer tests are also needed because responses to novelty, explanations, and feedback prompts may fade.

Statistical reports should quantify uncertainty, practical effect sizes, and repeated-seed variation where relevant. Public code and explicit preprocessing are particularly important because sessionization and exposure filtering can change results before training begins (Bernardis et al., 2022). Table 6 links recommended metric families to television-specific interpretations and minimum reporting requirements.

**Table 6:** Recommended evaluation families for smart TV content recommendation.

Family	Metrics	TV-specific interpretation	Reporting requirement
Top-k relevance	Precision@k, Recall@k, Hit Rate@k, NDCG@k, MAP@k, MRR	Whether relevant programmes appear early in a remote-navigated slate	Define relevance, candidate set, k, negative sampling, and confidence intervals.
Prediction/discrimination	MAE, RMSE, AUC, log loss	Rating error or discrimination under implicit labels	State label construction and exposure bias; avoid equating lower error with better slate utility.
Engagement	CTR, watch time, completion, return rate, zapping delay	Observed consumption and navigation cost	Report guardrails, novelty effects, time horizon, and whether outcomes are online.
Catalog value	Coverage, diversity, novelty, serendipity, calibration	Breadth and fit of a household-facing slate	Specify distance, popularity, expectation, and calibration definitions.
Household utility	Average, least-misery, disagreement, worst-member utility, identity accuracy	Whether recommendations serve multiple viewers and remain robust to identity error	Report group formation, aggregation rule, member-level outcomes, and sensitivity.
Responsibility	Exposure fairness, age suitability, privacy budget, explanation fidelity	Distribution of opportunity, child safety, data protection, and justified recommendations	Define stakeholders, protected groups, consent, and failure costs.
Systems	Latency, memory, energy, model size, refresh cost, uptime	Feasibility on TV devices and large catalogs	Separate training from serving cost and specify hardware and cache policy.

## 7 Foundation models and industrial systems

### 7.1 Multimodal and large language models

Foundation models can connect subtitles, synopses, dialogue, audio, frames, entities, and user language. This breadth may help with long-tail and cold-start items. Multimodal recommendation supplies transferable architectures (Liu et al., 2024; Malitesta et al., 2025), while xLiMe and Globoplay provide television-relevant cross-modal examples (Zhang et al., 2017; Ferreira et al., 2020). Recent work also considers LLMs for cross-domain recommendation and movie discovery (Liu et al., 2025; Raj et al., 2024).

Current evidence does not justify replacing retrieval and ranking pipelines with unconstrained generation. LLMs add latency and cost, can produce unstable or poorly calibrated outputs, and raise privacy concerns. More credible near-term roles include metadata normalization, semantic item representation, conversational query parsing, constrained reranking, and explanations grounded in verified catalog attributes.

Evaluation must separate fluent language from useful recommendation. Appropriate tests include strong sequential and multimodal baselines, grounding and hallucination errors, latency, cost, and robustness to household ambiguity. Explanations should remain tied to catalog facts and communicate uncertainty.

### 7.2 Evidence from deployed and industrial systems

Industrial evidence is uneven. COSMO reports an online A/B test on a commercial television service (Qin et al., 2023), and PLE increased views and watch time after deployment at Tencent (Tang et al., 2020). Globoplay evaluated multimodal similarity with users but left production A/B testing to future work (Ferreira et al., 2020). A separate industrial VoD study details data cleaning, session inference, and sequential baselines (Bernardis et al., 2022).

Across these systems, data engineering and interface constraints shape the observed algorithmic result. Session definitions, delayed labels, candidate availability, cache updates, and monitoring often determine whether an offline prototype becomes a stable service. Deployment conditions should therefore be treated as part of the scientific evidence. Table 7 contrasts demonstrated capability with the validation that remains necessary.

**Table 7:** Foundation-model and industrial evidence: demonstrated capability vs. remaining validation.

Area	Demonstrated evidence	Promising use	Required validation
Cross-modal semantics	Live-TV semantic annotation and industrial audiovisual similarity (Zhang et al., 2017; Ferreira et al., 2020)	Cold-start item embeddings and cross-lingual retrieval	Long-form temporal grounding, catalog drift, and online impact
LLM recommendation	Movie and cross-domain experiments (Liu et al., 2025; Raj et al., 2024)	Conversational discovery, semantic reranking, grounded explanations	TV logs, strong baselines, hallucination, latency, cost, and privacy
Household ranking	Commercial A/B evidence for coupling-aware modeling (Qin et al., 2023)	Uncertainty-aware role mixtures and manual correction	Member-level utility, guests, children, consent, and distribution shift
Multi-task optimisation	Large-scale online gains for PLE in video recommendation (Tang et al., 2020)	Balancing click, view, watch time, retention, and diversity	Household and public-value objectives; stable trade-offs
Industrial pipeline	Raw-log processing and session-aware VoD comparison (Bernardis et al., 2022)	Reproducible feature and monitoring contracts	Open protocols, cross-provider replication, and failure reporting
On-device/edge	Efficient session-based and TV-device approaches (Yin et al., 2025; Xia et al., 2023; Kim et al., 2018)	Private low-latency reranking and feedback handling	Energy, memory, update safety, and heterogeneous hardware

## 8 Research roadmap

Five research priorities emerge from the evidence, ordered by television specificity, scarcity of current support, and likely practical importance. Table 8 lists the corresponding near-term deliverables and the minimum evidence needed to judge progress.

**Table 8:** Prioritized research roadmap and minimum evidence needed for progress.

Priority	Research programme	Near-term deliverable	Minimum convincing evidence
1	Household identity uncertainty and group fairness	Open simulation protocol plus a privacy-preserving, overrideable role model	Identity and ranking ablations, member-level utility, robustness to wrong identity, consent analysis
2	Public TV benchmark and evaluation standard	De-identified session/EPG benchmark with availability, exposure, and household fields	Data statement, temporal splits, standard baselines, beyond-accuracy and systems metrics
3	Long-form multimodal and language models	Grounded item representations and conversational discovery with cached inference	Strong baselines, event-level grounding, hallucination audit, latency and cost
4	Multi-objective and trustworthy optimisation	Constraint-aware ranking across relevance, diversity, age suitability, fairness, and business value	Pareto analysis, calibrated trade-offs, explanations, privacy and safety guardrails
5	Deployment and longitudinal evaluation	Reference pipeline for sessionisation, monitoring, rollback, and feedback collection	Online or field testing, drift analysis, resource use, and reproducible preprocessing

### 8.1 Household-aware recommendation should model uncertainty

Future household systems should represent identity as uncertain rather than fixed. A distribution over possible viewers or groups would allow ranking to maximize expected utility while protecting the least-served member. Corrections made through the interface should update the current session without permanently relabelling household history.

Household models also need to limit raw sensing. Federated or on-device approaches may help (Chronis et al., 2024; Yin et al., 2025; Asad et al., 2023; Li X. et al., 2025; Xia et al., 2023), but credible evaluations must report communication costs, privacy budgets, hardware heterogeneity, and an explicit threat model.

### 8.2 Benchmarks must reproduce the television environment

A useful benchmark should record programme schedules, exposure positions, remote-control events, session gaps, content duration, and uncertainty about who is watching. It should also specify legal and ethical access conditions. General movie ratings may contribute to such a benchmark, but cannot alone establish performance in a smart-TV setting.

### 8.3 Foundation models should be modular and grounded

A modular architecture can use semantic models for candidate selection while keeping safety and business rules outside the generator. Catalog facts, age ratings, and current availability should constrain outputs. Lightweight or cached components are better suited to interactive latency.

### 8.4 Optimisation should expose trade-offs

Multi-objective systems should expose trade-offs through a Pareto frontier or sensitivity analysis. A single weighted score hides which stakeholder's objective is sacrificed. Household, platform, creator, advertiser, and public-service outcomes may require explicit constraints rather than one learned reward.

### 8.5 Deployment evidence should be treated as scientific evidence

Field studies should document model and interface versions, traffic allocation, novelty periods, guardrails, and stopping rules. Negative and neutral online results are informative because they show where offline ranking metrics fail to capture television use.

## 9 Limitations

Five limitations qualify the evidence map. First, its broad two-tier scope combines direct television studies with enabling work. Study-level transfer rationales and separate treatment of weak boundary evidence reduce, but do not remove, this heterogeneity. Second, because the search crossed television-domain terms with six prespecified challenge blocks, studies using only general recommendation terminology may have been less readily retrieved. Third, the inter-rater reliability assessment was conducted on a targeted 126-record eligibility validation cohort comprising all 120 included studies alongside six representative boundary exclusions, rather than the full title/abstract pool or a random sample of all excluded records. The resulting 97.6% agreement (κ = 0.788) therefore describes consistency within this audit set, not every screening stage. We checked the primary and secondary classifications and finalized them by consensus without calculating a separate category-specific reliability coefficient. Fourth, tasks, datasets, candidate sets, and metrics were too heterogeneous for quantitative meta-analysis, so incompatible performance results were neither pooled nor ranked.

The accompanying workbook contains the bibliographic corpus, the 36-query conceptual search framework and 216 executed database-native search strings, the six-category functional codebook, an anonymized full-text evidence-reporting profile, PRISMA-style corpus accounting, the six excluded boundary-control records and their reasons, the separate 126-record eligibility-audit contingency table, primary category distributions, and secondary technical-label frequencies and pairwise co-occurrences verified by us. Full-text evidence notes and source-specific search logs remain restricted internal records.

## 10 Conclusion

Smart TV content recommendation is defined by more than screen size. Passive interaction, shared identity, temporal availability, long-form content, and competing household and platform objectives distinguish the setting. Across 120 studies, the evidence map shows sustained methodological development but no universally dominant paradigm.

Traditional and hybrid approaches remain useful when data or hardware are limited. Deep, sequential, graph, and multimodal models offer richer representations, but evidence on cost, calibration, and transfer is still thin. Household-aware and industrial studies are promising, yet public benchmarks, member-level evaluation, and longitudinal online validation remain uncommon.

The next advance is less likely to come from another isolated accuracy gain than from closer alignment with household television use. Priorities include privacy-preserving household models, grounded representations of long-form content, explicit treatment of competing objectives, reproducible TV datasets, and evaluation of both system performance and user welfare.
